# Recent Advances in Thermoresponsive OEGylated Poly(amino acid)s

**DOI:** 10.3390/polym13111813

**Published:** 2021-05-31

**Authors:** Chao Geng, Shixue Wang, Hongda Wang

**Affiliations:** State Key Laboratory of Electroanalytical Chemistry, Changchun Institute of Applied Chemistry, Chinese Academy of Sciences, Jilin 130022, China; cgeng@ciac.ac.cn (C.G.); hdwang@ciac.ac.cn (H.W.)

**Keywords:** thermoresponsive, oligo(ethylene glycol), OEGylated, poly(amino acid), ring-opening polymerization, post-polymerization modification, Ugi reaction

## Abstract

Thermoresponsive polymers have been widely studied in the past decades due to their potential applications in biomedicine, nanotechnology, and so on. As is known, poly(*N*-isopropylacrylamide) (PNIPAM) and poly(oligo(ethylene glycol)methacrylates) (POEGMAs) are the most popular thermoresponsive polymers, and have been studied extensively. However, more advanced thermoresponsive polymers with excellent biocompatibility, biodegradability, and bioactivity also need to be developed for biomedical applications. OEGylated poly(amino acid)s are a kind of novel polymer which are synthesized by attaching one or multiple oligo(ethylene glycol) (OEG) chains to poly(amino acid) (PAA).These polymers combine the great solubility of OEG, and the excellent biocompatibility, biodegradability and well defined secondary structures of PAA. These advantages allow them to have great application prospects in the field of biomedicine. Therefore, the study of OEGylated poly(amino acid)s has attracted more attention recently. In this review, we summarized the development of thermoresponsive OEGylated poly(amino acid)s in recent years, including the synthesis method (such as ring-opening polymerization, post-polymerization modification, and Ugi reaction), stimuli-response behavior study, and secondary structure study. We hope that this periodical summary will be more conducive to design, synthesis and application of OEGylated poly(amino acid)s in the future.

## 1. Introduction

Stimuli-responsive polymers, so called “smart polymers” that can be triggered by a variety of external environmental stimuli such as temperature, pH, light, ionic, chemical and biological stimuli etc., and consequently with the change of physical and chemical properties, have been extensively investigated because of their potential applications in the past few decades [1,2,3,4,5,6,7,8,9,10,11,12]. Among them, thermoresponsive polymers, which exhibit a reversible phase transition to temperature, have attracted much attention due to their easy to control stimulus and potential biomedical and tissue engineering applications [13,14,15]. Thermoresponsive behaviors of polymers can be generally classified into two categories, lower critical solution temperature (LCST) type and upper critical solution temperature (UCST) type based on the equilibrium phase separation [16]. In both types, phase separation will occur and result in a turbid mixture of the two phases at a concentration dependent cloud point temperature (*T*_cp_), with *T*_cp_ ≥ LCST for separation with increasing temperature or *T*_cp_ ≤ UCST for separation with decreasing temperature, and a single phase for temperatures intermediate to these two regimes. Since the phase transition temperatures are closely dependent on the polymer structure (e.g., backbone, side-chain, topological architecture), it is very important to obtain the desired thermoresponsive temperature through reasonable structure design for specific applications. As is known, poly(*N*-isopropylacrylamide) (PNIPAM) is generally considered to be the gold standard because of its LCST around physiological temperature (≈32 °C) in water together with a low concentration and pH dependency, making it a prime candidate for in vivo biomedical applications [17,18]. Meanwhile, other types of polymers are increasingly being investigated for their thermoresponsive behavior in recent years; especially, polymers bearing an oligo(ethylene glycol) (OEG) side chain have been shown to combine the biocompatibility of OEG with a versatile and controllable LCST behavior, such as poly(oligo(ethylene glycol)methacrylate)s (POEGMA)s [19,20,21,22,23,24]. However, backbone of these thermoresponsive polymers are nondegradable, the negative effects of in vivo enrichment are unclear. As such, it is still necessary to search for novel thermoresponsive polymers with excellent biocompatibility, degradability and a tunable critical transition temperature to meet the needs of related fields.

OEGylated poly(amino acid)s are a kind of nonionic hydrophilic polymer obtained by covalently attaching one or multiple OEG chains to poly(amino acid) (PAA). These polymers combine the great solubility of OEG, and the excellent biocompatibility, biodegradability and well defined secondary structures of PAA (e.g., *α*-helix or *β*-sheet) [25,26,27,28,29]. The biggest advantage of this combination is that it overcomes commonly water soluble poly(amino acid)s (e.g., poly(l-glutamic acid), poly(l-glutamic acid)), which suffer from pH-dependent solubility and limited circulation lifetime because of their aggregation with oppositely charged polymers [29,30,31]. Although OEGylated poly(amino acid) is very important, its related research started late. Deming group reported the first OEGylated poly(l-lysine) (PLL), which showed excellent water-solubility and completely *α*-helical in solution in 1999 [30]. Then, they also synthesized methylated mono and di(ethylene glycol)-functionalized poly(L-serine) and poly(l-cysteine) [32]. Subsequently, Zhang and coworkers synthesized OEGylated poly(l-glutamic acid) encoding pendant alkyne side groups that were amendable to further modifications [33]. Inspired by these pioneer works, the OEGylated poly(amino acid)s were prepared as thermoresponsive materials with difference amino acids, and different OEG topological structures (e.g., linear, Y-shaped) and length; the expansion of structural diversity provides more possibilities for its applications.

This review summarizes the thermoresponsive OEGylated poly(amino acid)s over recent years (Table 1). Specifically, it focuses on the synthesis method, stimuli-response behavior study, and secondary structure study of OEGylated poly(amino acid)s. These will be discussed in the next two sections in detail. We hope that this periodical summary will be more conducive to the design, synthesis and application of OEGylated poly(amino acid)s in the future.

## 2. Synthetic Strategies of OEGylated Poly(amino acid)s

**Figure 1:**
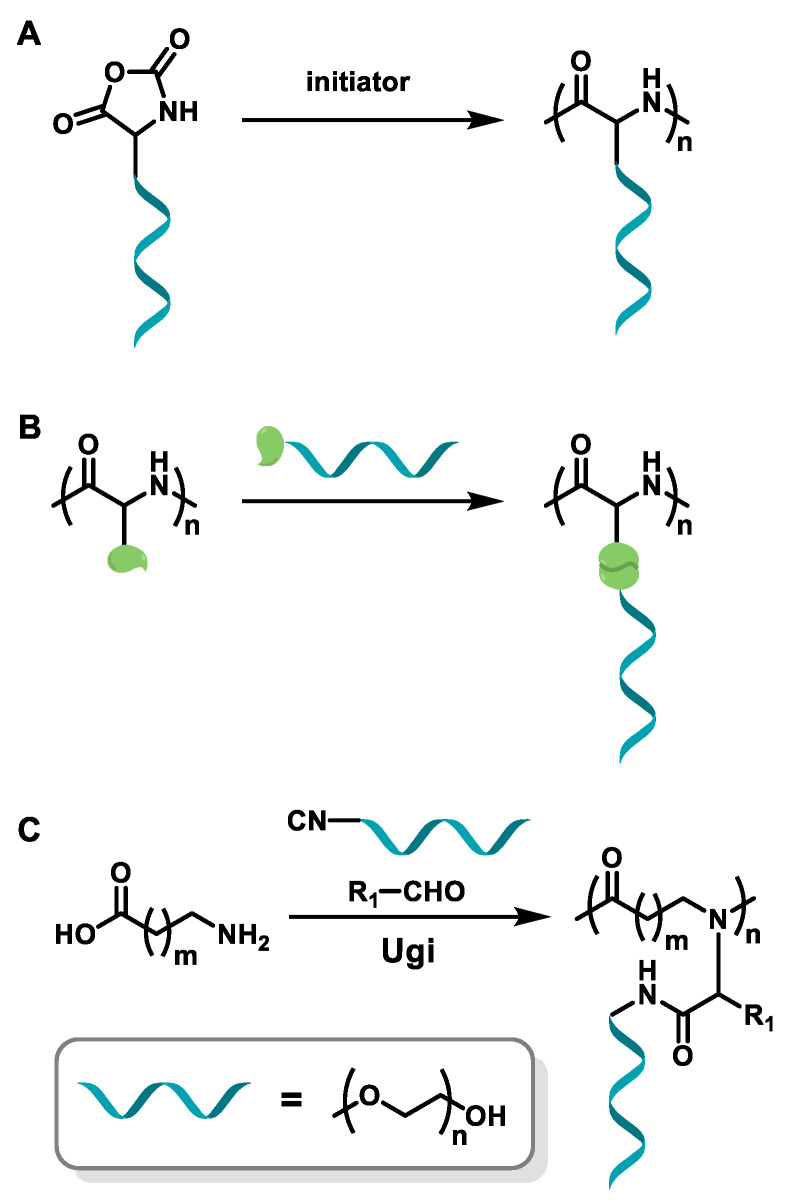
Synthesis strategies of OEGylated poly(amino acid)s. (**A**) ROP of OEGylated NCAs, (**B**) post-polymerization modification of poly(amino acid) precursors, and (**C**) Ugi multicomponent polymerization.

So far, OEGylated poly(amino acid)s can be generally synthesized by three ways: controlled ring-opening polymerization (ROP) of OEGylated *N*-carboxyanhydride (NCA) monomers, post-polymerization modification (PPM) of poly(amino acid) precursors and the Ugi multicomponent polymerization (Figure 1). These methods have their own advantages and disadvantages. The controlled ROP of OEGylated NCAs can obtain well-defined OEGylated poly(amino acid)s with controlled molecular weights (MWs), and are easy to scale up, which is beneficial for accurately exploring structure-property relationships and widespread applications. However, it has to take a lot of time to synthesize and purify the unstable OEGylated NCA monomers. In comparison, post-polymerization modification of poly(amino acid)s has the facility to construct OEGylated poly(amino acid)s with tunable OEG side-chain and properties, yet it need to use highly efficient reactions (usually the “click chemistry”) and sacrifice a large number of reactants to obtain a relatively perfect polymer structure. In recent years, multicomponent reactions (MCRs) such as the Passerini three-component reaction, Ugi reaction, Biginelli reaction, and so on, have drawn great attention and been utilized in the fields of polymer chemistry because of the mild reaction conditions, high efficiency, atom economy, and structural diversity of products [41,42,43,44]. Therefore, a series of polymers with a new backbone, side-chains, and topologies have been successfully prepared. An Ugi reaction is considered to be a flexible method to construct amide bonds using amine and carboxylic acid as the starting materials. Meier and coworkers firstly used this strategy to obtain polyamides with finely tunable structures with the diamine (AA monomer) and dicarboxylic acid (BB monomer) monomers [45]. From this background, Tao and coworkers have employed the natural amino acids as AB monomer and oligo(ethylene glycol) isocyanide and aldehyde as other two components to make sequence-specific OEGylated poly(amino acid)s, and studied the relationship of sequence structure and thermoresponsive behaviors [39,40,46]. This work provided a key example for the effect of sequence structure on thermoresponsive behaviors, which we will discuss in detail later. 

### 2.1. Thermoresponsive OEGylated Poly(amino acid)s from ROP of NCA Monomers

**Figure 2:**
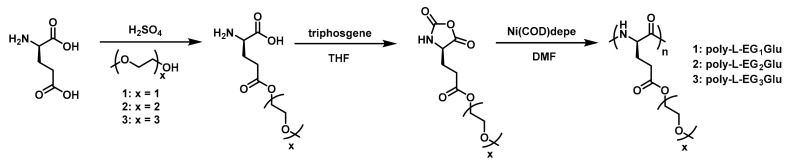
Synthetic route to poly-l-EGxGlu by Li et al. Reproduced with permission [34]. Copyright 2011, American Chemical Society.

In the past decades, NCA polymerization has developed rapidly; in addition to the traditional amine initiator [47], a few novel and efficient initiators have been developed, such as transition metal complex and hexamethyldisilazane (HMDS) [48,49,50,51]. This has helped to achieve better control of polymer structures and facilitate downstream material applications. In this context, several thermoresponsive OEGylated poly(amino acid)s which were synthesized via ROP of NCAs have been reported. In 2011, Li et al. firstly reported the synthesis and characterization of new thermoresponsive OEGylated poly-l-glutamate (poly-l-EG_x_Glu) (Figure 2) [34]. They synthesized the OEGylated NCAs via direct coupling between methylated ethyleneglycols and l-glutamate, then converted it into corresponding α-amino acid NCAs using triphosgene in THF. The obtained NCAs were viscous oils and purified by flash a column chromatography method. Then, different OEGylated homopolymers and random copolymers with narrow PDIs (<1.2) were prepared using Ni(COD)depe as initiator and DMF as solvent.

**Figure 3:**
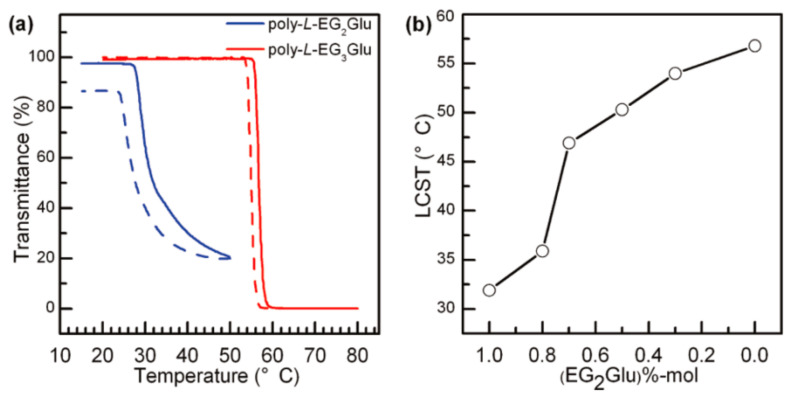
(**a**) Plots of transmittance as a function of temperature for aqueous solutions (2 mg/mL) of poly-l-EG_2_Glu and poly-l-EG_3_Glu. Solid line: heating; dashed line: cooling. (**b**) LCST of poly(EG_2_Glu-EG_3_Glu) copolymers as a function of sample composition. Reproduced with permission [34]. Copyright 2011, American Chemical Society.

OEGylated poly-l-EGxGlus obtained by Li et al. display reversible LCST transition in water, except poly-l-EG_1_Glu (insoluble in water), the LCST of poly-l-EG_2_Glu and poly-l-EG_3_Glu are 32 °C and 57 °C, respectively. They also found that both poly-l-EG_2_Glu and poly-l-EG_3_Glu displayed hysteresis in phase transition during cooling processes, which was probably due to the redissolution of the OEG unit, requiring slight overcooling to overcome the energy barriers (Figure 3a). It is worth noting that it is easy to make random OEGylated copoly(amino acid)s with different EG_2_Glu/EG_3_Glu ratios in the Ni(COD)depe catalytic system with nanarrow molecular weight distribution (<1.2); meanwhile, the LCST can be varied from 36 °C with 80 mol% l-EG_2_Glu to 54 °C with 30 mol% l-EG_2_Glu (Figure 3b). It is noteworthy that the physiological temperature is just in this LCST range. 

**Figure 4:**
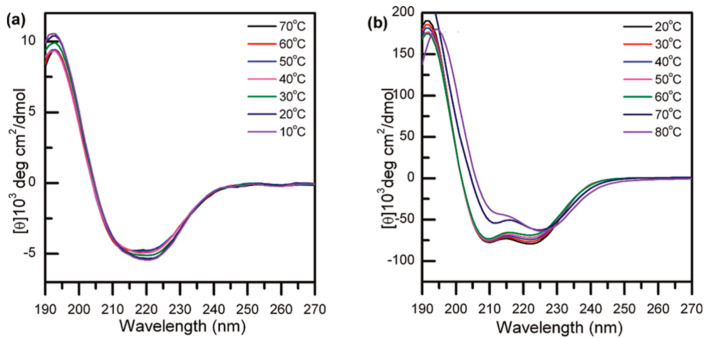
CD spectra of (**a**) poly-l-EG_2_Glu and (**b**) poly-l-EG_3_Glu as a function of temperature (heating scan). Reproduced with permission [34]. Copyright 2011, American Chemical Society.

Subsequently, Li et al. studied the secondary structure of poly-l-EG_x_Glus in water using circular dichroism (CD) spectra. Poly-l-EG_2_Glu purified by dialysis did not have a well-defined secondary structure, which was composed of 16% α-helix, 32% *β*-strand, 20% turns, and 32% random coil, respectively. However, it almost formed 100% α-helix in freshly prepared aqueous solution. Heating the same solution above its LCST did not cause an obvious change in corresponding secondary structures (Figure 4a); it indicated that the secondary structure of poly-l-EG_2_Glu strongly depended on sample history. In contrast, poly-l-EG_3_Glu formed stable 100% α-helix and its secondary structure was also independent of temperature (Figure 4b). These results reveal that the secondary structure of poly-l-EG_x_Glus is OEG chain length dependent. The longer OEG side-chain is beneficial to the stability of α-helix.

**Figure 5:**
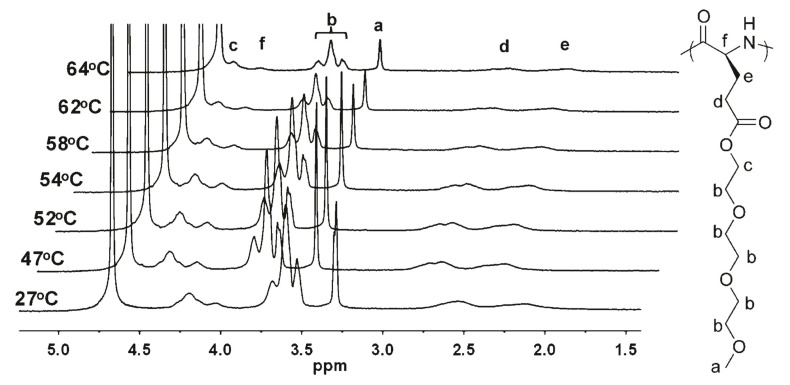
^1^H NMR spectra of poly-l-EG_3_Glu as a function of temperature in D_2_O. Reproduced with permission [34]. Copyright 2011, American Chemical Society.

Finally, Li et al. studied the driving force of LCST behaviors of poly-l-EGxGlu via temperature-dependent ^1^H NMR (Figure 5). With the increase in temperature, they found that the protons of end methoxy and methylene groups of OEG units became more and more broad, accompanying a substantial decrease in signal intensity; a further increase above their corresponding LCST caused almost disappearance of their resonances. These results indicated that temperature increase induced dehydration of ethylene glycol groups and caused the phase separation. 

The work of Li et al. developed a facile and economic strategy to prepare biodegradable thermoresponsive OEGylated poly(amino acid)s with narrow molecular weight distribution. The LCST of these materials can be tuned by changing the length of OEG units. CD characterization suggested that the secondary structures of poly-l-EGxGlus relied on the chain length of OEG side chains. These thermoresponsive poly(amino acid)s with tunable LCST will have great promise to construct new intelligent biomaterials for biomedical applications.

**Figure 6:**
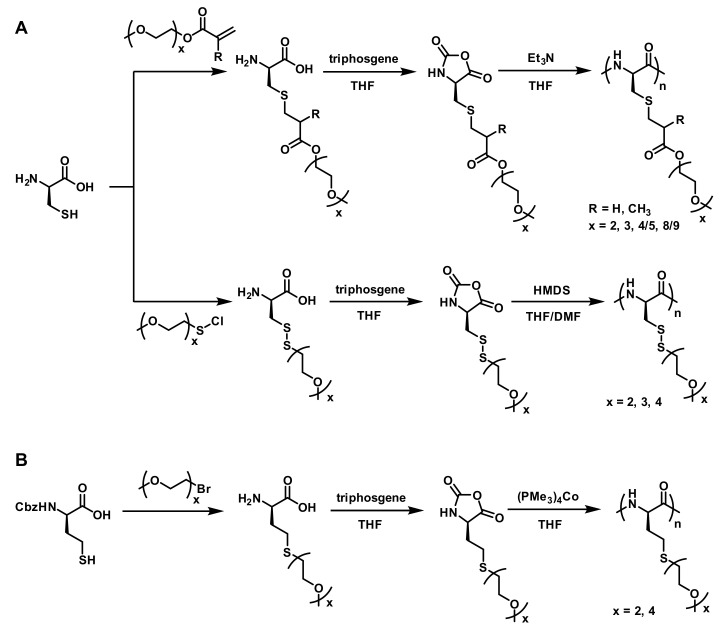
Synthetic routes to OEGylated poly(amino acid)s from cysteine/homocysteine by (**A**) Li’s group [35,36], and (**B**) Deming’s group [28].

**Figure 7:**
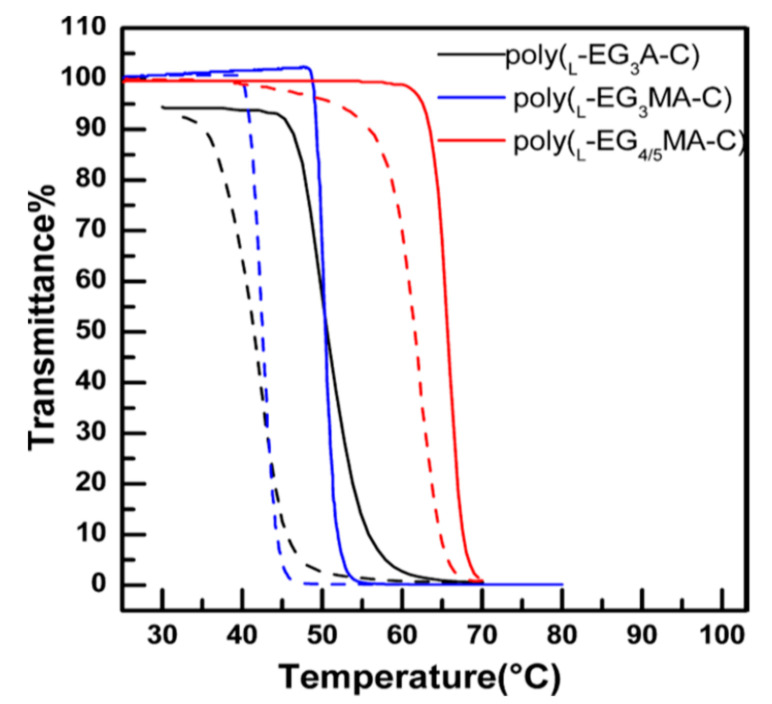
Plots of transmittance as a function of temperature for aqueous solutions (2 mg/mL) of (poly-l-EG_3_MA-C), (poly-l-EG_4/5_MA-C), and (poly-l-EG_3_A-C). Solid line: heating; dashed line: cooling. Reproduced with permission [35]. Copyright 2013, American Chemical Society.

The above work shows a successful example for constructing OEGylated poly(amino acid)s as thermoresponsive materials. Therefore, it is of particular interest and importance to further develop novel and highly efficient methods to make thermoresponsive OEGylated poly(amino acid)s from easily available chemicals. Cysteine/homocysteine are amino acids with a thiol group; thiol is more easily derivatized by thiol-ene Michael addition or nucleophilic substitution reactions, because of their extremely good nucleophilicity. In this context, a series of novel OEGylated poly(amino acid)s were synthesized by Li’s group and Deming’s group using cystine and homocysteine as starting materials (Figure 6). In 2013, Li et al. reported a series of new functional amino acids which were prepared via thiol-ene Michael addition between l-cysteine and OEG functionalized methacrylates (OEG_x_MA) or acrylate (OEG_x_A) in a high yield [35]. These OEGylated cysteine derivatives were converted into NCA monomers using triphosgene. Subsequently, triethylamine (Et_3_N) was used to catalyze the ROP of these NCA monomers to give a series of OEGylated poly-l-cysteines (poly-EG_x_MA-C or poly-EG_x_A-C) (Figure 6A). The resulting poly-EG_x_MA-C and poly-EG_x_A-C displayed OEG length dependent solubility and secondary structure in water. More importantly, when the x value is between three and five, the obtained polymers can display reversible thermoresponsive properties in water, such as poly-EG_3_A-C, poly-EG_3_MA-C, and poly-EG_4/5_MA-C, the LCSTs are 50 °C, 65 °C, and 51 °C (Figure 7), respectively. The synthetic strategy represents a highly efficient method to prepare OEGylated poly(amino acid)s with tunable thermoresponsive properties.

**Figure 8:**
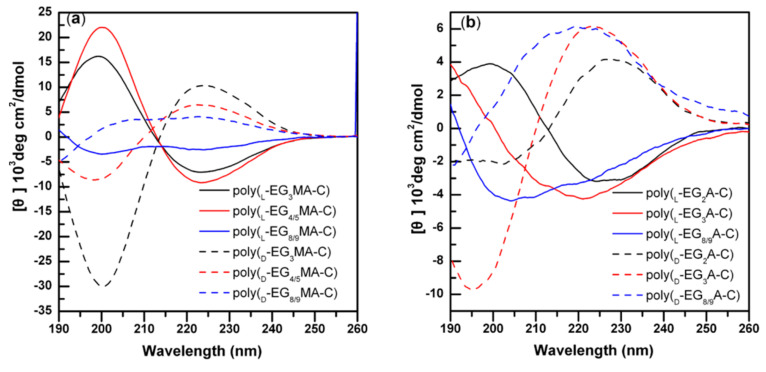
CD spectra of different OEGylated polycysteine homopolypeptides: (**a**) poly-l-EG_x_MA-C (solid line) and poly-d-EG_x_MA-C (dashed line); (**b**) poly-l-EG_x_A-C (solid line) and poly-d-EG_x_A-C (dashed line). Reproduced with permission [35]. Copyright 2013, American Chemical Society.

It was known that poly-l-cysteine was a *β*-sheet forming polypeptide [52]; previous studies showed that conjugation of di(ethylene glycol)thioester to the poly-l-cysteine side chain did not disrupt its *β*-sheet conformation [32]. However, poly-l-cysteine conjugated with hydrophilic sugars adopted helical conformation [53]. In this report, Li et al. also investigated the effects of OEG side chain length on the secondary structures of poly-l-cysteine derivatives using CD spectroscopy. In Figure 8, the results revealed that both series of samples formed mixed conformation, in which a random coil was the major conformation. This result does not agree with the previous reports; the authors had analyzed that there were two possible reasons for the mixed secondary conformation. One of these was that the synthetic method of poly-l-EG_x_MA-C or poly-l-EG_x_A-C made them have longer side chains than poly-l-EG_x_Glu, and the long side chain could destabilize the stability of secondary structure. Another possible reason was that the MWs were not high enough, which might seriously affect the content of secondary conformation. Li’s report provides a new reference for secondary structures of poly-l-cysteine derivatives.

**Figure 9:**
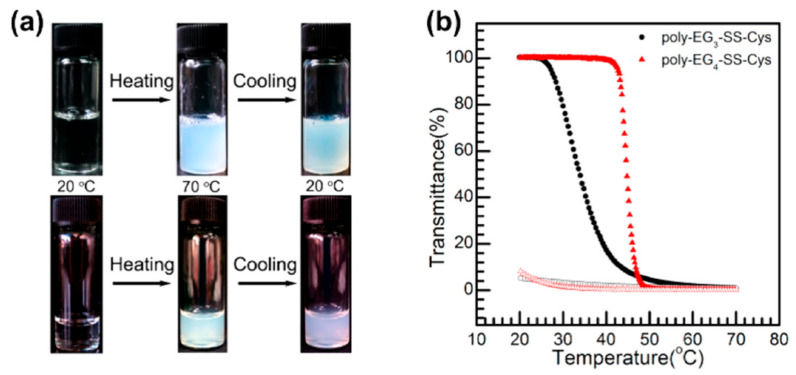
(**a**) Photos of temperature induced phase transition for poly-EG_3_-SS-Cys (top) and poly-EG_4_-SS-Cys (bottom) aqueous solutions at 2 mg/mL. (**b**) Transmittance as a function of temperature for aqueous solutions (2 mg/mL) of poly-EG_3_-SS-Cys (black) and poly-EG_4_-SS-Cys (red). Solid symbols: heating ramp. Open symbols: cooling ramp. Reproduced with permission [36]. Copyright 2014, American Chemical Society.

Furthermore, Li et al. also synthesized three cysteine derivatives in high yields by ligating OEG to thiol group of l-cysteine using sulfenyl chlorides [36]. These OEG groups containing di-, tri-, and tetra-OEG units were linked with l-cysteine via disulfide bonds. The three monomers were then converted into corresponding NCAs, and subsequently poly-EG_x_-l-cysteines via ROP with HMDS as catalyst (Figure 6A). The obtained poly-EG_x_-l-cysteine with x = 3 and 4 displayed thermoresponsive behaviors in water, but the temperature-induced phase transition was found to be surprisingly irreversible (Figure 9). Such irreversible thermoresponsive behaviors were attributed to cross-linking arising from disulfide bonds exchanges. Using PEG-NH_2_ as macro-initiator, they also prepared two PEG-*b*-poly-EG_x_-l-cysteine diblock copolymers, which could undergo irreversible thermal-induced sol-gel transition. These hydrogels displayed partially shear-thinning and rapid recovery properties, allowing new capabilities to construct stimuli-responsive injectable hydrogels in biomedical applications.

**Figure 10:**
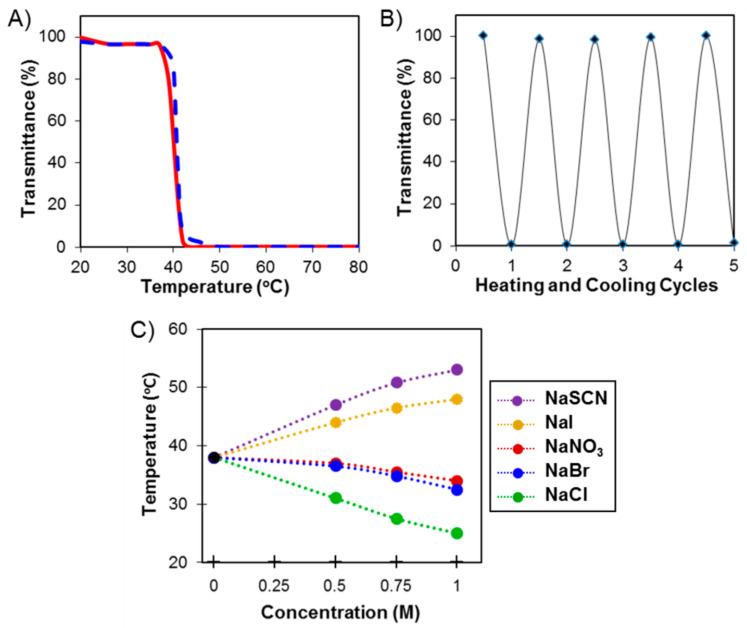
(**A**) Influence of temperature on light transmittance (500 nm) through a sample of aqueous poly(OEG_4_-CH)_150_. Solid red line = heating; dashed blue line = cooling; 1 °C/min. (**B**) Reversible change in optical transmittance of aqueous poly(OEG_4_-CH)_150_ when temperature was alternated between 30 °C (high transmittance) and 45 °C (low transmittance); 5 min per each heating/cooling cycle. (**C**) Cloud point temperatures of poly(OEG_4_-CH)_150_ measured in different Hofmeister salts (Na^+^ counterion) at concentrations up to 1.0 M. All poly(amino acid)s were prepared at 3 mg/mL. Reproduced with permission [28]. Copyright 2014, American Chemical Society.

In 2014, Deming et al. reported the design and synthesis of poly(*S*-alkyl-l-homocysteine)s through ROP of homocysteine derived NCAs (Figure 6B). These are a new class of readily prepared, multi-responsive polymers that possess the unprecedented ability to respond to different stimuli, either through a change in conformation or in water solubility [28]. Among them, heating aqueous samples of poly(OEG_4_-CH)_150_, sharp transitions from clear solutions to opaque suspensions were observed, indicating the presence of LCST for these OEGylated poly(amino acid)s (Figure 10A). These transitions were completely reversible and could be repeated multiple times with no observable persistent precipitation or other changes to the sample (Figure 10B). In addition, authors also studied the thermoresponsive properties in the presence of different Hofmeister anions in detail, since anions are known to affect thermoresponsive properties of polymers more than cations (Figure 10C). The effects of different salt concentrations on the LCST of poly(OEG_4_-CH)_150_ followed trends similar to those seen with other thermoresponsive polymers, and allow tuning of the transition temperature [54,55]. The thermoresponsive properties of OEGylated poly(l-homocysteine)s, combined with their potential adjustability, makes them promising candidates for a broad range of stimuli responsive material challenges.

**Figure 11:**
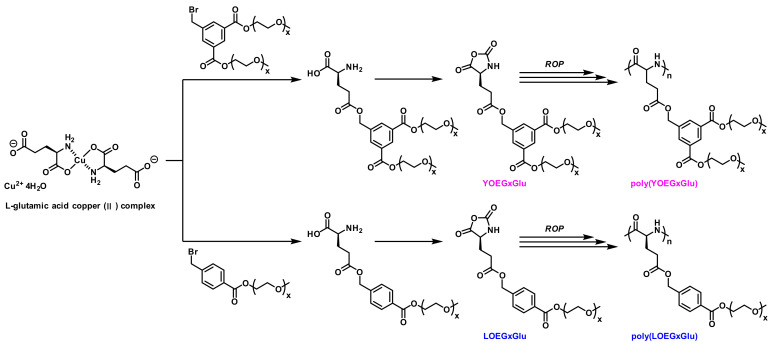
Synthesis of linear and Y-shaped OEGylated poly(YOEG_x_Glu)s and poly(LOEG_x_Glu)s via NCA polymerization. Reproduced with permission [27]. Copyright 2019, American Chemical Society.

**Figure 12:**
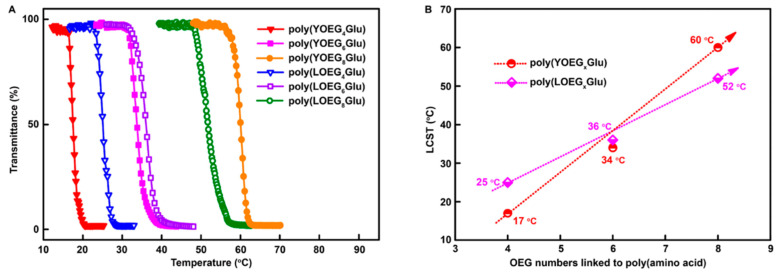
(**A**) Profiles of transmittance vs temperature for the aqueous solutions (2 mg/mL) of poly(LOEG_x_Glu)s and poly(YOEG_x_Glu)s. (**B**) LCSTs of poly(LOEGxGlu)s (◆) and poly(YOEGxGlu)s (●); x represents the number of OEG units. Reproduced with permission [27]. Copyright 2019, American Chemical Society.

**Figure 13:**
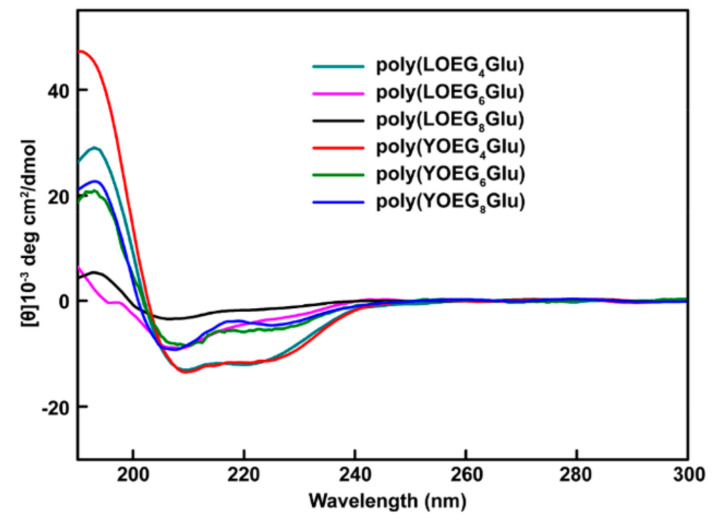
CD spectra of poly(LOEG_x_Glu)s and poly(YOEG_x_Glu)s measured in CH_3_CN at 25 °C (c = 0.5 mg/mL). Reproduced with permission [27]. Copyright 2019, American Chemical Society.

Despite the crowning achievements in linear OEGylated poly(amino acid)s and manipulation of the properties, exhaustive understanding of the topological architecture of OEG side-chains remains a work in progress. The distinctive topological architecture has distinct properties from their linear analogues, such as solubility, viscosity, and so forth [56]. In 2019, Tao et al. designed and synthesized a series of new linear and Y-shaped OEGylated poly(glutamic acid)s (Figure 11). They have systematically characterized and compared the thermoresponsiveness and secondary structures of several poly(glutamic acid) conjugates including linear and Y-shaped OEGs [27]. The results revealed that the LCST of OEGylated poly(glutamic acid)s could be turned by the length of OEG numbers. More importantly, the LCST of OEGylated poly(glutamic acid)s was firmly correlative to the OEG architecture (Figure 12). For example, the LCST of the Y-shaped poly(YOEG_8_Glu) was higher than that of its linear analogue poly(LOEG_8_Glu) (e.g., 60° for poly(YOEG_8_Glu) and 52° for poly(LOEG_8_Glu)). This observation is consistent with what one would forecast based on the steric repulsion influence, as the Y-shaped OEGylated polypeptides are more sterically congested than linear OEG because of the dense pendants, which would lead to a greater extent of hydration shell and thus higher LCST. Indeed, steric repulsion may result in the elevated LCST, as already demonstrated by Bitton [56]. However, it appears that this effect is OEG length-dependent and grows pronounced only when the number of the OEG units is ≥6. Notably, the Y-shaped OEGylated poly(glutamic acid)s exhibit higher α-helical conformation than linear ones (Figure 13), which is critically essential in respect to constructing nonionic water-soluble poly(amino acid)s with stable secondary structures. Collectively, this contribution not only provides an appealing route toward Y-shaped OEGylated poly(amino acid)s, but also affords us abundant knowledge to understand how the OEG architecture interferes with the performances of poly(amino acid)s.

### 2.2. Thermoresponsive OEGylated Poly(amino acid)s from Post-Polymerization Modification

**Figure 14:**
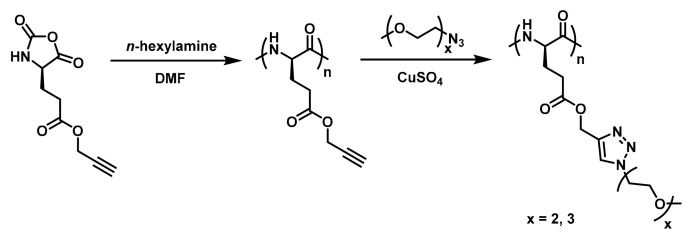
Synthesis route of the OEG graft poly(l-glutamate) by Chen’s group. Reproduced with permission [37]. Copyright 2011, Royal Society of Chemistry.

**Figure 15:**
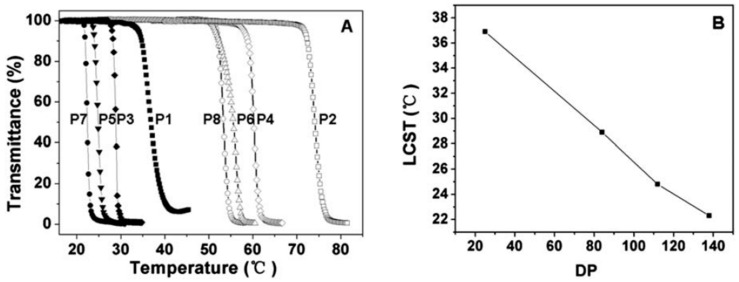
(**A**) Thermal phase transitions of P1 (PPLG_25_-*g*-MEO_2_), P2 (PPLG_25_-*g*-MEO_3_), P3 (PPLG_84_-*g*-MEO_2_), P4 (PPLG_84_-*g*-MEO_3_), P5 (PPLG_112_-*g*-MEO_2_), P6 (PPLG_112_-*g*-MEO_3_), P7 (PPLG_138_-*g*-MEO_2_) and P8 (PPLG_138_-*g*-MEO_3_), and the polymer concentration was 10 g/L. (**B**) The LCSTs of PPLG-*g*-MEO_2_ as a function of the degree of polymerization. Reproduced with permission [37]. Copyright 2011, Royal Society of Chemistry.

Post-polymerization modification of poly(amino acid)s is a facility method to construct OEGylated poly(amino acid)s with tunable OEG side-chain length and properties. “Click chemistry” is undoubtedly the most suitable method. In this respect, Chen’s group firstly synthesized a series of novel alkyne functionalized poly(l-glutamic acid) via ROP of alkyne functionalized NCAs. Subsequently, the pendant alkyne groups coupled with 1-(2-methoxyethoxy)-2-azidoethane (MEO_2_-N_3_) or 1-(2-(2-methoxyethoxy)ethoxy)-2-azidoethane (MEO_3_-N_3_) by the efficient azide-alkyne “Click chemistry” to obtain OEGylated poly(amino acid)s (Figure 14) [37]. These were named PPLG_n_-*g*-MEO_x_. The graft copolymers exhibited sharp temperature dependent phase transitions, and the LCST could be adjusted from 22.3 °C to 74.1 °C by varying the molecular weight and the length of the OEG side chains (Figure 15). In addition, these OEG graft poly(amino acid)s were confirmed to be biocompatible and non-toxic using the methyl thiazolyl tetrazolium (MTT) method, and they were degradable in the presence of proteinase K. Drug loading and release was also conducted with these thermosensitive nanoparticles using doxorubicin as the model drug, and a temperature-dependent sustained release profile was observed. Therefore, it is believed that these novel OEG graft poly(l-glutamic acid)s with tunable temperature responsiveness should be promising for smart biomedical applications.

**Figure 16:**
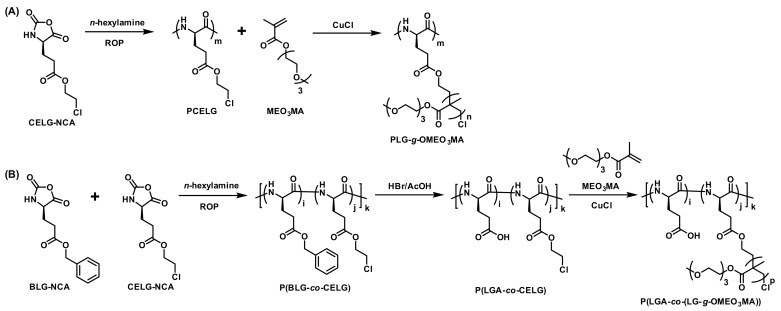
Synthetic routes of copolymers (**A**) PLG-*g*-OMEO_3_MA and (**B**) P(LGA-*co*-(LG-*g*-OMEO_3_MA)) by Chen et al. Reproduced with permission [38]. Copyright 2011, Wiley Periodicals, Inc.

Thermoresponsive and pH-responsive graft co-polymers, PLG-*g*-OMEO_3_MA and P(LGA-*co*-(LG-*g*-OMEO_3_MA)), were also synthesized by Chen and coworkers though ROP of NCA monomers and subsequent atom transfer radical polymerization of 2-(2-(2-methoxyethoxy)ethoxy)ethyl methacrylate in 2011 (Figure 16) [38]. The thermoresponsive of OEG graft co-polymers could be tuned by the MWs of OMEO_3_MA, the composition of poly(l-glutamic acid) (PLGA) and the pH of the aqueous solution. The α-helical contents of graft copolymers could be influenced by OMEO_3_MA length and pH of the aqueous solution. In addition, the graft copolymers exhibited tunable self-assembly behavior. The hydrodynamic radius (*R*_h_) and critical micellization concentration values of micelles were relevant to the length of OMEO_3_MA and the composition of the biodegradable PLGA backbone. The *R*_h_ could also be adjusted by the temperature and pH values. Lastly, in vitro MTT assay revealed that the graft copolymers were biocompatible to HeLa cells. Therefore, these graft copolymers with good biocompatibility, well-defined secondary structure, and mono- dual-responsiveness, are promising stimuli-responsive materials for biomedical applications.

### 2.3. Thermoresponsive OEGylated Poly(amino acid)s from Ugi Multicomponent Polymerization

**Figure 17:**
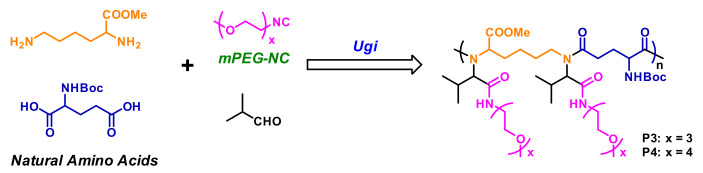
Synthesis of alternating OEGylated poly(amino acid)s via Ugi reaction of natural amino acids. Reproduced with permission [39]. Copyright 2018, American Chemical Society.

**Figure 18:**
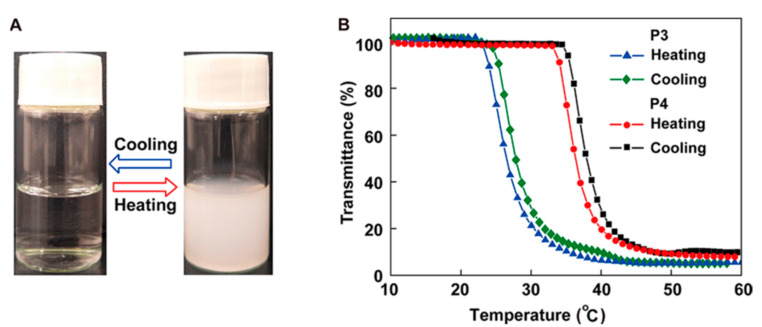
(**A**) Visual turbidity change of P4 upon heating the aqueous solution. (**B**) Temperature dependence of transmittance for the aqueous solutions (2 mg/mL) of P3 and P4 (500 nm, heating or cooling at a rate of 1 °C/min). Reproduced with permission [39]. Copyright 2018, American Chemical Society.

Ugi reaction is a four-component reaction, and the reactants are commonly acid, amine, isocyanide, and aldehyde [57,58]. This reaction has gained great attention and been utilized in the fields of combinatorial chemistry, pharmaceutics, and life science due to its mild reaction conditions, high efficiency, functional group tolerance, and atom economy [59,60]. Recently, Meier demonstrated a very efficient and modular approach to synthesizing diversely substituted polyamides via the Ugi four-component reaction [45]. In contrast to conventional polyamide synthesis, this approach proceeds under very mild reaction conditions and without the use of a catalyst in a one-pot reaction. Subsequently, Tao and coworkers reported the synthesis of structurally diverse poly(amino acid)s (also called polypeptoids) by Ugi polymerization of natural amino acids under mild conditions, and this strategy offered a general methodology toward facile preparation of functionalized poly(amino acid)s [46]. Based on this work, Tao et al. also designed and synthesized a series of new alternating poly(amino acid)s via the Ugi reaction of readily available natural amino acids. Among them, the thermoresponsive OEGylated poly(amino acid)s have been prepared using oligo(ethylene glycol) isocyanide (Figure 17), and exhibited cloud points (*T*_cp_) between 27 °C and 37 °C (Figure 18). The alternating structure and diverse polymer properties described here offer a new direction for the synthesis of novel OEGylated poly(amino acid)s materials [39].

**Figure 19:**
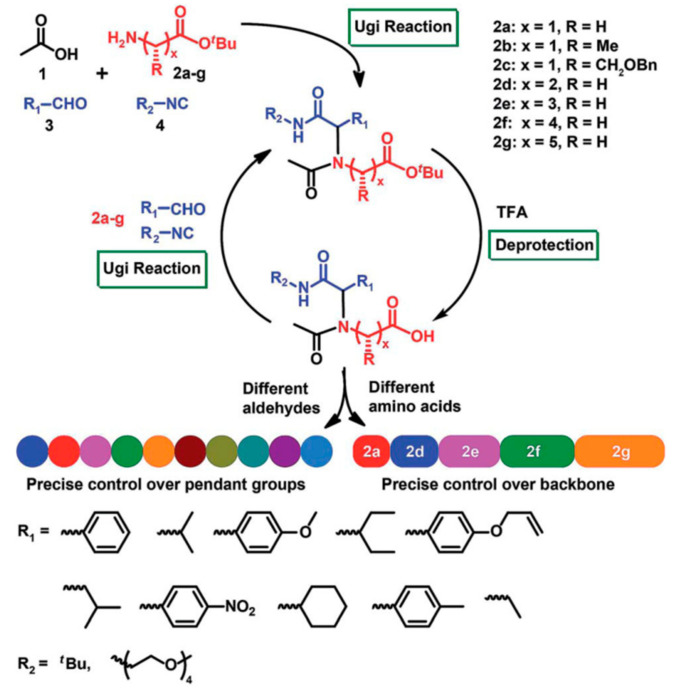
Synthesis strategy towards sequence-defined poly(amino acid)s via amino acid building blocks and iterative Ugi reactions. Reproduced with permission [40]. Copyright 2019, Royal Society of Chemistry.

**Figure 20:**
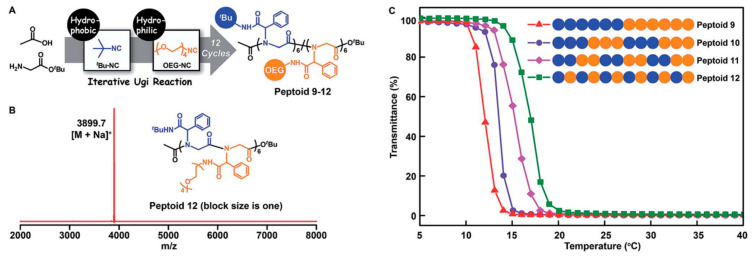
(**A**) Illustration of the side-chain sequence-regulated OEGylated poly(amino acid)s 9–12 synthesized by iterative Ugi reactions. (**B**) MALDI-TOF-MS spectrum of 12. (**C**) Temperature dependence of transmittance for the aqueous solutions (2 mg/mL) of sequence-regulated OEGylated poly(amino acid)s 9–12 (500 nm, heating at a rate of 1 °C/min). Reproduced with permission [40]. Copyright 2019, Royal Society of Chemistry.

Recently, sequence-controlled synthetic polymers have been drawing great interest because the specific sequences can endow more advanced functions to the polymers, such as DNA and proteins [61,62,63,64,65]; however, the control remains a great challenge in polymer science, and this drives people to find more concise and efficient methods to construct polymers with accurate sequence structure. Additionally, the molecular functions and properties determined by the sequence structure are less studied. In this context, Tao et al. firstly reported the development of amino acid building blocks coupled with iterative Ugi reactions for the efficient and multigram-scale assembly of sequence-defined poly(amino acid)s (Figure 19) [40]. This efficient chemistry provides much feasibility for structural diversity, synthetic varying and sequencing both the side chains and the backbones. Using this advanced method, they coupled the OEG units in the sequence-defined polymers, and further demonstrated that the alteration in the overall hydrophobicity and LCST behaviors of these precisely defined OEGylated poly(amino acid)s could be accordingly changed by variation of the sequence (Figure 20). Regulation of sequence-specific hydrophobic aggregation within a polymer is a significant result. This versatile strategy may afford new materials for application in therapeutics, and as supramolecular foldamers or simple protein mimics for the investigation of advanced self-assembly driven by hydrophobic or other supramolecular interactions.

## 3. Applications

Applications of thermoresponsive polymers have been studied in many fields, such as biomedicine, tissue engineering, and in sensors [15,66,67]. For example, PNIPAM, as the most widely studied thermoresponsive polymer, has been extensively used in many biomedical applications. Andrew and coworkers loaded the insulin on PNIPAM based thermoresponsive microgels, and studied the release using variable temperature ^1^H NMR [68]. This type of direct release investigation could prove to be a useful method in the future design of controlled macromolecule drug delivery devices. Additionally, thermoresponsive polypeptides and polypeptoids also show a good application prospect. Lu et al. synthesized a series of thermoresponsive polypeptides incorporated with various functional side groups [69]. They found that these polypeptides showed perfect hemostatic properties and healing effects which are expected to be potential candidates for medical applications, such as tissue adhesives. Zhang and coworkers demonstrated that water-soluble horseradish peroxidases can be easily encapsulated in a thermoresponsive triblock copolypeptoid hydrogels for extended period of time with the retained enzymatic activity, and the hydrogels show low cytotoxicity towards human adipose-derived stem cells [70]. These results indicate the potential utilization of the polypeptoid hydrogel as tissue engineering material.

For thermoresponsive OEGylated Poly(amino acid)s, little application research has been conducted. Chen et al. evaluated the cytotoxicity of graft copolymers PPLG_112_-g-MEO_2_ in vitro by MTT assay [37]. It was observed that the HeLa cells treated with PPLG_112_-g-MEO_2_ remained almost 100% viable at all test concentrations up to 1 mg/mL, indicating non-cytotoxicity and good biocompatibility of the graft copolymers. Then, they investigated the temperature-dependent drug release behavior of the drug-loaded co-polymer nanoparticles, and doxorubicin (Dox) was used as a model drug. The results suggested that the drug release from the temperature-sensitive amphiphilic nanoparticles could be accelerated by increasing the temperature above their LCSTs. In addition, the release of Dox displayed a constant rate in the first 24 h at 37 °C. This demonstrated that an ideal constant Dox release could be obtained in the nanoparticle system. The result suggested that the graft co-polymers could be promising candidates as drug carriers for controlled drug delivery.

## 4. Conclusions and Outlook

Thermoresponsive OEGylated poly(amino acid)s combined the advantages of OEG and poly(amino acid)s with great solubility, excellent biocompatibility and well-defined secondary structures. These advantages allow it to have great application prospects in the field of biomedicine, tissue engineering, and sensors. However, it is still in the initial research stage. In this review, we summarized the research progress of thermoresponsive OEGylated poly(amino acid)s in recent years, including the synthesis methods, stimuli-response behavior study, and secondary structure study of these OEGylated poly(amino acid)s. We hope that this periodical summary will be more conducive to the design, synthesis and application of OEGylated poly(amino acid)s in the future.

In addition, the design of the structure and function for practical applications is the future development direction of OEGylated poly(amino acid)s; it is suggested to carry out targeted research in the following aspects. In respect to polymer design, the topological structure of side-chains (such as cyclic, star-shape) need to be expanded to bring about the diversity of structures and functions. Moreover, it is necessary to select the appropriate amino acids according to the secondary structure requirements of OEGylated poly(amino acid) materials. In respect to the synthesis method, although ROP of NCAs and the post-polymerization modification are the typical methods to construct OEGylated poly(amino acid)s, some novel strategies need to be developed for efficient preparation of the sequence-defined polymers, such as the Ugi multi-component polymerization. Finally, its application in the field of medicine should be further strengthened due to its great application prospect.

## Figures and Tables

**Table 1:** Summary of thermoresponsive OEGylated poly(amino acid)s.

Entry	Polymer Structure	Amino Acids	Synthetic Method	*T*_cp_ (°C)	Secondary Structure	Ref.
1	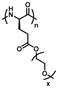	l-Glutamic acid	ROP	x = 2, *T*_cp_ = 32 °C x = 3, *T*_cp_ = 57 °C	x = 2, 100% α-helix in freshly prepared aqueous solution x = 3, 100% helix	[34]
2	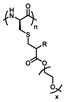	l-Cysteine	ROP	R = CH_3_, x = 3, *T*_cp_ = 50 °C x = 4, *T*_cp_ = 65 °C R = H, x = 3, *T*_cp_ = 51 °C	α-helix, β-sheet, and random coil	[35]
3	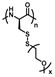	l-Cysteine	ROP	x = 3, *T*_cp_ = 34 °C x = 4, *T*_cp_ = 45 °C	α-helix, β-sheet, and random coil	[36]
4	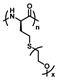	l-Homocysteine	ROP	x = 4, *T*_cp_ = 40 °C	>95% α-helix	[28]
5	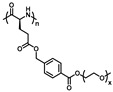	l-Glutamic acid	ROP	x = 4, *T*_cp_ = 25 °C x = 6, *T*_cp_ = 36 °C x = 8, *T*_cp_ = 52 °C	x = 4, 69% α-helix x = 6, 29% α-helix x = 8, 17% α-helix	[27]
6	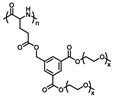	l-Glutamic acid	ROP	x = 2, *T*_cp_ = 17 °C x = 3, *T*_cp_ = 34 °C x = 4, *T*_cp_ = 60 °C	x = 2, 69% α-helix x = 3, 37% α-helix x = 4, 30% α-helix	[27]
7	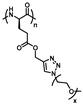	l-Glutamic acid	Post-polymerization modification	x = 2 or 3, *T*_cp_ = 22.3–74.1 °C by varying the molecular weight	100% α-helix	[37]
8	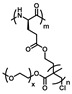	l-Glutamic acid	Post-polymerization modification	x = 3, *T*_cp_ = 45.7–51.3 °C by varying the molecular weight	78.4–100% α-helix	[38]
9	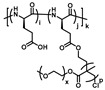	l-Glutamic acid	Post-polymerization modification	x = 3, *T*_cp_ = 44.1–62.1 °C by varying the molecular weight and monomer ratio	0–65.1% α-helix	[38]
10	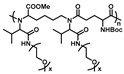	l-Lysine and l-Glutamic acid	Ugi multicomponent polymerization	x = 3, *T*_cp_ = 27 °C x = 4, *T*_cp_ = 37 °C	random coil	[39]
11	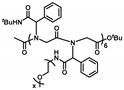	Glycine	Ugi multicomponent polymerization	x = 4, *T*_cp_ = 12–17.5 °C with difference sequence	random coil	[40]

## Data Availability

Not applicable.
